# Unlocking Seed Dormancy and Elucidating Storage Behavior in *Morinda royoc* (Rubiaceae): Crucial Insights for Propagation and Ex Situ Germplasm Conservation

**DOI:** 10.3390/biology15100770

**Published:** 2026-05-12

**Authors:** Duniel Barrios, Ricardo Álvarez-Espino

**Affiliations:** 1 Planta!—Plantlife Conservation Society, Vancouver, BC V2X7R6, Canada; 2 Laboratorio Regional para el Estudio y Conservación de Germoplasma (GermoLab), Centro de Investigación Científica de Yucatán (CICY), Parque Científico Tecnológico de Yucatán, km. 5.5 Carretera Sierra Papacal-Chuburná Puerto, Mérida 97302, Mexico; 3 Secretaría de Ciencia, Humanidades, Tecnología e Innovación (SECIHTI), Mexico City, Mexico

**Keywords:** germination, seed morphology, scarification, piña ch’oom, garañón, cheese-shrub

## Abstract

*Morinda royoc* L. is a shrub with high pharmacological value due to its antimicrobial and metabolic bioactivity. However, its low germination limits its commercial use and conservation. This study characterized the morphophysiology of its diaspores, evaluated pre-germination treatments (mechanical endocarp removal and GA_3_ soaking at 600 and 1200 ppm), and determined its storage behavior. The analyses showed a highly significant effect where manual endocarp removal overcame mechanical restriction and eliminated dormancy, achieving 100% germination within two weeks, whereas the control reached only 51.5% after 23 weeks. Treatments with GA_3_ did not improve germination compared to the control, and an inhibitory effect was observed at 1200 ppm, although 100% of the non-germinated embryos remained viable. The seeds maintain viability above 90% after three months of storage at 25, 5, and −20 °C. Our findings suggest that *M. royoc* seeds exhibit orthodox behavior (tolerant to desiccation and freezing) and non-deep physiological dormancy. This study documents, for the first time, a method for the complete release of dormancy in the species, which is essential for successful propagation. Our results provide a rapid and economical germination method that facilitates mass propagation, establishing the technical basis for the transition toward commercial-scale cultivation and ensuring the effective conservation of *M. royoc* germplasm.

## Simple Summary

*Morinda royoc* is a shrub highly valued for its medicinal properties, including antimicrobial and potential anticancer activities. Despite its importance, commercial cultivation is limited by poor germination, leading to costly cloning or unsustainable wild harvesting. In this study, we investigated the seed characteristics and tested various methods to overcome this lack of germination. We discovered that the main obstacle is the hard outer layer of the seed (endocarp), which physically prevents growth. By simply removing this layer, we achieved 100% germination in just two weeks. Additionally, we found that the seeds can survive long-term storage at freezing temperatures. These findings establish an efficient and accessible germination protocol for *M. royoc*, contributing to its ex situ conservation and supporting its sustainable industrial use by facilitating seed-based plant production while reducing the pressure on wild populations.

## 1. Introduction

*Morinda royoc* L. is a vine-like shrub of the Rubiaceae family with recognized medicinal properties [1]. It is commonly known as piñón de monte, piña ch’oom (Mexico), piñipiñi, garañón (Cuba), and cheese-shrub (USA) [2,3,4]. Its distribution spans the Yucatán and Florida peninsulas (Mexico and the U.S.), much of the insular and continental Caribbean, and Mesoamerica and northern South America [4,5]. The species grows in a range of habitats, from coastal and dry ecosystems to evergreen forests [5]. Due to its wide distribution, it is considered a species of Least Concern [6]; nevertheless, Jiménez et al. [7] suggest that indiscriminate harvesting for medicinal use could threaten the species in the future.

Historically, *Morinda royoc* has been highly valued in traditional medicine for treating a wide range of conditions, including menstrual disorders, respiratory problems, dysentery, jaundice, and sexual dysfunction, in addition to its use as a purgative and laxative [4]. In Mexico, the Maya culture of the Yucatán Peninsula attributes various ethnobotanical uses to it [8]. The roots are used for stomach pain and cancer or applied as a poultice for varicose veins [8,9]. The fruit or its juice is used topically to remove warts [8]. Other Mayan uses include its function as a diuretic, astringent, and treatment for kidney and liver problems and snake bites [8,10].

Based on traditional knowledge, numerous studies on *M. royoc* have demonstrated significant bioactivity. Extracts from its roots are rich in anthraquinones such as morindone and soranjidiol, with morindone being the main active compound [8,11]. This bioactivity includes a potent effect against *Giardia lamblia* [8] and broad-spectrum antimicrobial activity. Regarding its antimicrobial profile, dichloromethane root extracts have been shown to be potent against various strains of *Candida* and *Staphylococcus aureus* [11]. The essential oil from the fruit, rich in octanoic acid, has also shown high activity against *C. albicans* and *C. utilis* [10]. In general, all plant extracts (leaves, fruit, and root) have shown activity against *Escherichia coli* and *S. aureus* [12]. Furthermore, activity has been demonstrated against plant pathogens such as *Xanthomonas campestris* and *Rhizoctonia solani* [13], including a preventive effect comparable to synthetic fungicides in vitro on pineapple plants [14].

Despite the significant pharmacological interest in *M. royoc* extracts, very little information is available regarding its propagation, and most studies have relied on wild-harvested plants [7,10,11,15,16,17]. This underscores the urgent need for research to enable large-scale cultivation [1,7] and long-term seed conservation. According to Jiménez et al. [1], low seed viability has been reported as the main limiting factor for both natural and commercial propagation. A fundamental step in understanding these propagation difficulties involves knowledge of its fruit and seed morphology. In this species, the fruits are short drupes grouped into a syncarp; thus, the structure usually recognized as a ‘fruit’ is actually an infructescence (approximately 2 cm long), whereas the ‘seed’ is a diaspore comprising the true seed and the lignified endocarp [4]. Chemical or mechanical scarification of the endocarp, or embryo stimulation using phytohormones such as GA_3_, could potentially help break seed dormancy and promote germination, as previously reported for *M. citrifolia* [18,19,20]. However, to date, no germination studies have evaluated these treatments in *M. royoc*.

Recently, Linares [14] reported well-developed spatulate embryos for *M. royoc*; although the authors did not achieve germination in a four-week trial, they reported a viability of over 73% for their seeds. This lack of germination in seeds with well-developed embryos and endocarps with marginal fissures [4] suggests physiological dormancy in *M. royoc*, ruling out morphological or morphophysiological dormancy [14]. Baskin and Baskin [21], in their review of dormancy classes for Rubiaceae, specifically report that, for the tribe Morindae, orthodox seeds exhibit no dormancy, physiological dormancy, or morphophysiological dormancy, thereby ruling out the presence of physical or combined (physical + physiological) dormancy for the family.

Given the aforementioned challenges, understanding the morphophysiological characteristics of the seeds and studying the germination requirements of *M. royoc* are essential for establishing effective propagation and conservation strategies [14] and constitute critical factors for the mass production of seedlings [19]. Therefore, this study aims to overcome these limitations by (1) characterizing the morphophysiological traits of *M. royoc* seeds; (2) evaluating their germination response to different pre-germination treatments; and (3) assessing their storage behavior. We hypothesize that *M. royoc* has orthodox seeds that require pre-germination treatments to break dormancy.

## 2. Materials and Methods

### 2.1. The Species

*Morinda royoc* L. is an erect or vine-like shrub, sometimes climbing to a height of 6 m with flexuous or twining branches. Leaves are linear-lanceolate to oblong-lanceolate, 4.5–12.5 cm long and 1–4.5 cm wide, acute to slightly acuminate at the apex, with a cuneate to acute base, and a papery to cartaceous texture. Inflorescences are globose, 0.5–1 cm in diameter, with peduncles 0.3–1.5 cm long. The corolla is white to pinkish, approximately 8 mm long, with briefly exserted anthers [22,23]. The fruit is a globose, yellow drupaceous syncarp, 0.8–2.5 cm in diameter [24], aggregated from multiple ovaries; endocarps (pits) sometimes exhibit marginal fissures and contain a single floating seed [4].

### 2.2. Collection Site

Fifty-one *M. royoc* fruits were collected on 25 July 2025, in the Yucatan Peninsula (Dos Aguadas, Quintana Roo, Mexico; 18.1046° N, −89.1066° W). The site is located at an elevation of 141 m above sea level within a medium-sized deciduous forest. Annual precipitation is 1339 mm, and the average annual temperature is 24.9 °C [25]. Fieldwork and biological material collection were authorized under permit No. SBRA/DGVS/01863/25, issued by the Dirección General de Vida Silvestre of the Subsecretaría de Biodiversidad y Restauración Ambiental (SEMARNAT, Ciudad de Mexico, Mexico).

### 2.3. Morphological and Functional Characterization

Due to the development of *M. royoc* infructescences, the structure referred to as a ‘seed’ comprises both the seed and the endocarp [4]. We refer to the unit containing the endocarp and the seed as a diaspore; therefore, the names of the traits measured may differ from those used in previous studies (e.g., [14]). Infructescences were stored at room temperature until the fourth day after collection, when diaspores were cleaned with a sieve and running water. Measurements of seed traits were taken three days after cleaning.

Seed traits were measured on two sets of 25 randomly selected diaspores. The following traits were evaluated for the first set of diaspores: length, width, thickness, fresh mass, dry mass, moisture content, endocarp (coating) mass, seed mass, and seed-coat endocarp:diaspore ratio (SCR). For the second set, seed length, endosperm length, embryo length, embryo:seed ratio, and embryo:endosperm ratio were measured. Measurements for the first set were taken with a digital caliper (±0.01 mm), while the second set was measured using calibrated images obtained with a Motic stereoscope (version 1.0.19). Mass values were determined using an analytical balance (±0.0001 g). Dry mass was obtained by drying the diaspores in an oven at 100 °C for 24 h to a constant weight, following modified FAO and ISTA [26] standards. Moisture content was calculated using the following formula:

(1) $$\mathrm{Moisture}\;\mathrm{content}\;\left(\%\right)\;=\;\frac{\mathrm{fresh}\;\mathrm{mass}\;-\;\mathrm{dry}\;\mathrm{mass}}{\mathrm{fresh}\;\mathrm{mass}}\;\times \;100$$

The SCR was determined by manually separating the endocarp from dry diaspores using a scalpel and forceps.

### 2.4. Germination Experiments

The germination of *M. royoc* seeds was evaluated using three pre-germination treatments and a control. The first treatment consisted of the mechanical removal of the endocarp. The following two treatments involved soaking the diaspores for 24 h in GA_3_ at 600 and 1200 ppm, respectively. Finally, in the control group, diaspores were soaked in distilled water for the same duration. Prior to imbibition, diaspores were disinfected as follows: 1 min in a 0.2% commercial detergent solution, 1 min in 98% alcohol, and 5 min in 2% sodium hypochlorite, with distilled water rinses between each step.

For each treatment, 105 diaspores were used (7 replicates of 15 diaspores each), except for the endocarp removal treatment, which consisted of 4 replicates due to an insufficient number of seeds. Diaspores were placed in Petri dishes (60 × 15 mm) with filter paper and 20 mL of distilled water. The dishes were sealed with Parafilm to prevent moisture loss and placed in a growth chamber at 30 °C, 80% relative humidity, and a 12/12 h light/dark photoperiod (300 lum/ft^2^). The experiment was checked weekly for 18 weeks and concluded at week 23. Germination was defined by a visible radicle length of at least 1 mm.

Viability of non-germinated seeds was assessed using a 0.5% 2,3,5-triphenyl tetrazolium chloride (TZ) solution dissolved in distilled/deionized water, for 24 h at 30 °C in darkness. For this analysis, 10 non-germinated seeds were randomly selected from each treatment. Before staining, the endocarp was removed and a longitudinal incision was made to expose the embryo, following the general principles for forest and shrub seeds established by FAO and ISTA [26]. The staining pattern of each embryo was examined individually under a stereomicroscope; seeds were considered viable when the embryo stained completely red.

### 2.5. Storage Behavior

To determine storage behavior, 7.7054 g of *M. royoc* seeds were placed in a mosquito-net bag inside a desiccator with silica gel at room temperature. Seeds were weighed weekly until reaching moisture content of 5% [27]. The final mass was divided into three parts and stored in plastic containers with cotton and silica gel for one and three months at 25, 5, and −20 °C. Initial viability was assessed using the TZ protocol described above. Post-storage viability was assessed via germination tests (with endocarp removal), using 25 seeds per temperature (5 replicates of 5 seeds), and monitored weekly for eight weeks.

### 2.6. Statistical Analysis

Germination data were analyzed using a Generalized Linear Model (GLM) with a binomial distribution and a logit link function. To evaluate the overall effect of the treatments, an Analysis of Deviance was performed using the Likelihood Ratio Test. Multiple comparisons between treatment means were conducted using Tukey’s test (α = 0.05) based on the model’s estimated marginal means.

Furthermore, to evaluate the effects of temperature and storage time on seed germination, an additional GLM with a binomial distribution and logit link function was employed. Additive and interaction models were compared using the Akaike Information Criterion (AIC), selecting the most parsimonious model for final interpretation. The significance of these factors was determined through an Analysis of Deviance. Model adequacy was assessed by comparing residual deviance with degrees of freedom to confirm the absence of overdispersion.

All statistical analyses were performed in R (version 4.4.1, R Core Team) within the RStudio interface (version 2025.05.1). The base stats package was used for GLM fitting and Analysis of Deviance. Comparisons of estimated marginal means and Tukey post hoc contrasts were performed using the emmeans package, while the assignment of significance letters for treatment categorization was handled with the multcomp package [28]. Figures were generated in SigmaPlot 10.0 and refined in Adobe Photoshop CS3 (version 10.0).

## 3. Results

### 3.1. Characterization of the Seeds

**Figure 1:**
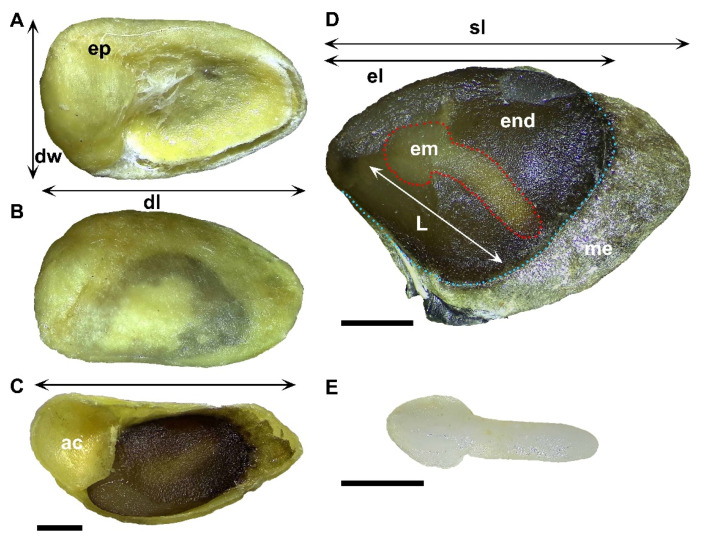
Diaspore and seed traits of *Morinda royoc*. (**A**–**C**): diaspore; (**D**): seed; (**E**): embryo. ep: endocarp; dw: diaspore width; dl: diaspore length; ac: air chamber; sl: seed length; end: endosperm; el: endosperm length; em: embryo; L: embryo length; me: membrane. Scale bars = 1 mm.

**Figure 2:**
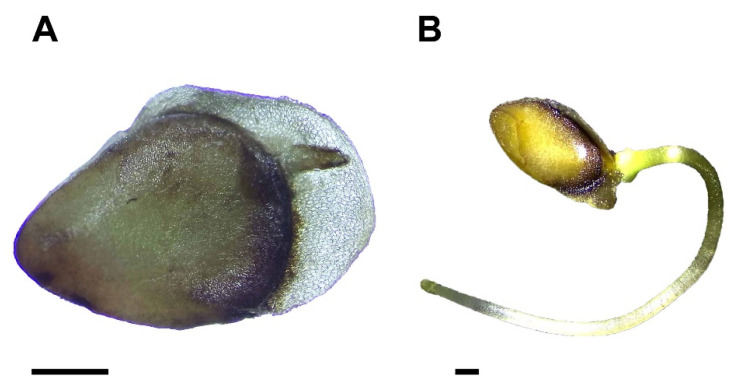
Germination of *Morinda royoc* seeds. (**A**): radicle breaking through the seed coat; (**B**): germinated seed one week after sowing. Scale bars = 1 mm.

The diaspores of *M. royoc* are characterized by a stony endocarp that protects the true seeds (Figure 1A–C). These seeds possess a thin, brown testa which extends into a distinct membranous wing or sail (Figure 1D). During the elongation process, the radicle penetrates this structure (Figure 2A,B). Internally, the *M. royoc* embryos are white and exhibit a spatulate morphology that ranges from straight to markedly curved, or occasionally nearly linear (Figure 1D,E). Detailed quantitative morphological traits, including dimensions, mass, and ratios for both diaspores and seeds, are summarized in Table 1.

### 3.2. Germination and Viability

**Figure 3:**
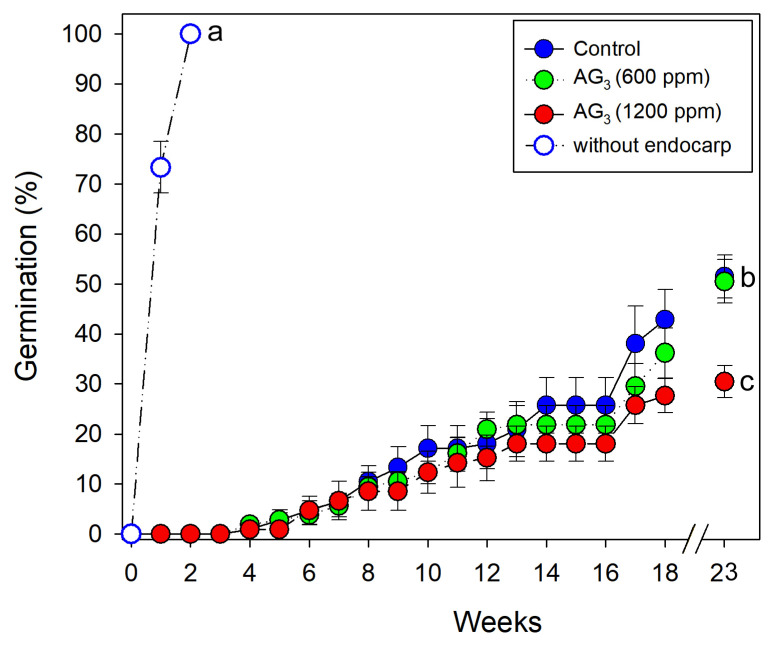
Cumulative germination of *Morinda royoc* under three pre-treatments and a control (mean ± *SE*, *n* = 7). Different letters indicate significant differences between treatments (α = 0.05).

Germination of *M. royoc* significantly differed among treatments (D = 97.50, df = 3, *p* < 0.001). Seeds achieved 100% germination within two weeks only in the treatment where the endocarp was removed (Figure 2B and Figure 3). The GA_3_ treatments and the control group initiated germination at very low rates during the fourth week. For these treatments, the final germination rate after 23 weeks was below 55%, with the 1200 ppm GA_3_ treatment showing the lowest germination values (Figure 3). All non-germinated seeds remained viable.

### 3.3. Storage Behavior

**Figure 4:**
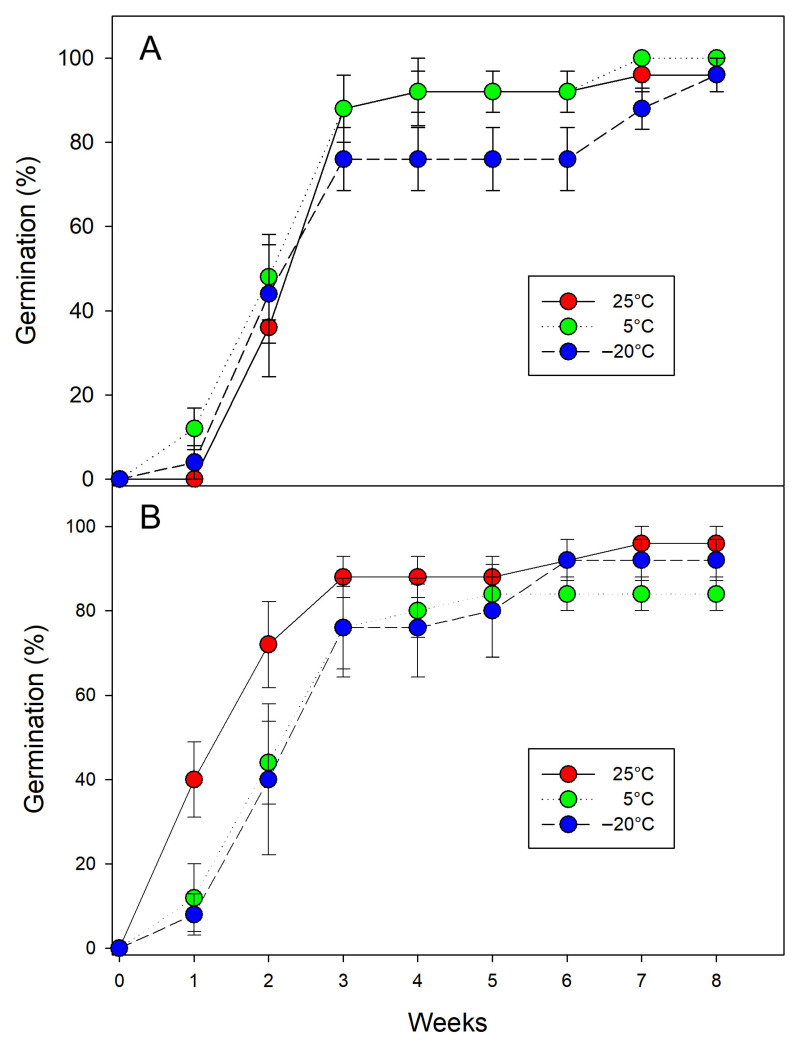
Cumulative germination of *Morinda royoc* seeds (**A**): after one and (**B**): three months of storage at different temperatures (mean ± *SE*, *n* = 5).

The diaspores reached a moisture content of 5% by the sixth week. The viability of seeds dehydrated prior to storage was 100%. Diaspores stored for one and three months under various conditions achieved germination rates exceeding 90%, with the exception of those stored at 5 °C for three months (Figure 4). However, no significant differences were found regarding temperature (D = 0.72, df = 2, *p* = 0.696), storage time (D = 3.13, df = 2, *p* = 0.076), or their interaction (D = 3.12, df = 2, *p* = 0.210). Non-germinated seeds were confirmed to be viable.

## 4. Discussion

While the dimensions of *Morinda royoc* seeds, endosperm length, and moisture content align with previous reports [14], our findings reveal significant morphophysiological variations. Notably, the fresh mass recorded in our study was approximately twice as high as in previous reports, despite the diaspores exhibiting smaller overall physical dimensions. A notable difference was found in embryo length; our specimens were 20% shorter, consistently measuring under 3 mm. These discrepancies in fresh mass and embryo size, including the embryo-to-endosperm ratio, may be driven by contrasting environmental pressures. Linares et al. [14] studied a population originating from a coastal xeromorphic scrub, whereas our study focused on plants from a medium-sized deciduous forest. This suggests that habitat-specific conditions significantly shape the morphophysiological profile of *M. royoc* seeds. Furthermore, the presence of developed spatulate embryos, and occasionally, nearly linear ones, coincides with reports in the family by Baskin and Baskin [21]. Linares et al. [14] also observed both types, although they specifically mentioned only the developed spatulate ones, regardless of whether they were straight or curved.

The delayed germination of *M. royoc* diaspores clearly indicates seed dormancy. According to the classification system of Baskin and Baskin [29] and reports for the tribe Morindae [21], non-deep physiological dormancy is the class that best fits this species. This class of dormancy aligns with the results obtained by Singh and Rai [20] when they treated *M. citrifolia* dispores—which are similar to *M. royoc* diaspores [30]—with various concentrations of GA_3_. However, our results showed no effect of GA_3_ on *M. royoc* germination, as has been observed in other Rubiaceae species [31,32], despite using the same concentrations as Singh and Rai [20]. In fact, germination was inhibited at the 1200 ppm dose. This inhibitory response is consistent with the mechanism proposed by da Silva et al. [33] for *Coffea arabica*, a species belonging to the Rubiaceae family, like *M. royoc*. According to these authors, an excess of exogenous gibberellins accelerates the hydrolysis of endosperm mannans, releasing toxic levels of mannose that interfere with ATP synthesis and cellular metabolism. In our study, the fact that 100% of the embryos remained viable (according to the TZ test) suggests that this GA_3_ concentration induced a metabolic block or hormonal stress without reaching lethal necrosis. However, further physiological studies are needed to confirm whether the mannose toxicity mechanism described in coffee is directly responsible for the inhibition observed in *M. royoc*.

Furthermore, the presence of a slit in the endocarp of *Morinda royoc* (Figure 1A), through which water penetrates the seed, rules out physical dormancy. Moreover, this class of dormancy has not been reported in the Rubiaceae family [21]. The previously reported physical dormancy in *M. citrifolia* [19] may require re-evaluation, as the observed response may reflect a misinterpretation of the release of physiological dormancy following in diaspores that were scarified using sulfuric acid and hot water. These treatments are also widely recommended for species with physiological dormancy [29,34,35]. According to Baskin and Baskin [35], it is not uncommon for researchers to incorrectly report physical dormancy in a particular taxon. They add: *“Almost without exception in such studies, lack of water uptake was not documented by comparing imbibition in scarified versus non-scarified seeds.”* This is precisely the case in the study by Ponnaiyan and Vezhavendan [19].

Our results showed that the mechanical constraint of the endocarp is the main impediment to *M. royoc* germination, as its removal was the only treatment that broke dormancy. According to Baskin and Baskin [29,35], the presence of fruit coats is one factor that can cause shallow physiological dormancy. Ponnaiyan and Vezhavendan [19] demonstrated that scarifying the endocarps of *Morinda citrifolia* with sulfuric acid softened them and increased germination.

To the best of our knowledge, this study is the first report of a method to achieve complete dormancy release in *M. royoc* diaspores. Historically, most pharmacological studies have used samples collected from wild populations in Cuba [7,10,11,15,16,17], Mexico [8], and Ecuador [12]. According to Borroto et al. [36] and Jiménez et al. [1], *M. royoc* diaspores exhibit low viability and sporadic germination. Consequently, Jiménez et al. [7] focused their efforts on in vitro vegetative propagation, a more costly technique that reduces genetic crop variability [37,38]. Therefore, our results provide the scientific community with a highly efficient germination protocol, a critical step for the successful propagation and initiation of sustainable, commercial-scale cultivation of this species. This will generate interest in its industrial exploitation.

Finally, given that *Morinda royoc* seeds showed tolerance to desiccation and freezing, they could be considered “potentially orthodox” according to the criteria of Hong and Ellis [27]. However, it is necessary to verify this prediction through long-term evaluation of germplasm quality under freezing conditions and to conduct studies across a larger number of populations within the species distribution range. This finding aligns with the work of Baskin and Baskin [21], who classified most Rubiaceae species as having seeds that are tolerant of dehydration and cold storage. Linares et al. [14] predicted orthodox or intermediate behavior based on the moisture content of the diaspores. Therefore, our study is the first to demonstrate the suitability of this storage method for *M. royoc* seeds in germplasm banks. This method provides a low-cost, highly efficient alternative compared to more expensive or complex preservation techniques. These findings provide a baseline for a technical guarantee for the long-term ex situ conservation of this species by ensuring that genetic material can be efficiently preserved for future pharmacological and ecological restoration projects.

## 5. Conclusions

Our study demonstrated that removing the endocarp breaks dormancy in *Morinda royoc* seeds. In contrast, exogenous application of GA_3_ (600 and 1200 ppm) failed to promote germination. In fact, the highest concentration had an inhibitory effect, likely due to a metabolic blockade or hormonal stress, although embryo viability remained at 100%. These results suggest that the primary germination constraint in this species is mechanical rather than hormonal. Additionally, we found that the seeds exhibit a probable orthodox behavior because they can be stored under cold conditions and low humidity.

## Figures and Tables

**Table 1:** Quantitative traits of *Morinda royoc* diaspores and seeds.

Diaspore/Seed	Traits	Mean ± SD
Diaspore	Length (mm)	5.94 ± 0.39
	Width (mm)	4.24 ± 0.45
	Thickness (mm)	2.15 ± 0.50
	Fresh mass (mg)	14.04 ± 2.85
	Dry mass (mg)	12.20 ± 2.45
	Moisture content (%)	12.99 ± 1.74
	Endocarp mass (mg)	7.36 ± 1.31
	Endocarp mass:dry mass	0.61 ± 0.07
Seed	Seed Length (mm)	4.84 ± 0.47
	Endosperm Length (mm)	3.78 ± 0.36
	Embryo Length (mm)	2.51 ± 0.27
	Embryo:Seed Ratio	0.52 ± 0.05
	Embryo:Endosperm Ratio	0.67 ± 0.07
	Mass (mg)	4.84 ± 1.38

## Data Availability

The original contributions presented in this study are included in the article. Further inquiries can be directed to the corresponding author.
